# Factors that influence breast cancer screening among women of reproductive age in the Nandom Municipality, Ghana

**DOI:** 10.1186/s12905-022-01946-0

**Published:** 2022-08-31

**Authors:** Margaret Mary Wuur, Dillys Adomakoa Duodu, Elvis Enowbeyang Tarkang

**Affiliations:** 1 Department of Population and Behavioural Sciences, School of Public Health, University of Health and Allied Sciences, Ho, Ghana; 2 HIV/AIDS Prevention Research Network Cameroon, Kumba, Cameroon; 3 Department of Public Health Medicine, School of Nursing and Public Health, University of KwaZulu-Natal, Durban, South Africa; 4 Department of Epidemiology and Biostatistics, School of Public Health, University of Health and Allied Sciences, Ho, Ghana

**Keywords:** Breast cancer screening, Women of reproductive age, Health belief model, Nandom municipality, Ghana

## Abstract

**Background:**

In Ghana, breast cancer is a major public health concern and the most common type of cancer among women in terms of mortality and incidence. This study determined the factors influencing breast cancer screening among women of reproductive age in Nandom Municipality, Ghana using the Health Belief Model as the conceptual model.

**Methods:**

The study was cross-sectional in design. A pretested structured questionnaire was administered to 243 womens of reproductive age in the Nandom Municipality. Descriptive and inferential statistics were performed using STATA version 16 at a 0.05 level of significance.

**Results:**

The uptake of breast cancer screening was 51.9%. Respondents who had a tertiary level of education were less likely to be screened for breast cancer [AOR = 0.10 (95% CI = 0.02–0.54); p = 0.008]. Respondents who perceived high susceptibility to breast cancer were more likely to get screened [AOR = 1.97 (95% CI = 1.12–3.47), p = 0.019]. Respondents who perceived the high severity of breast cancer were more likely to be screened for breast cancer [AOR = 4.55 (95% CI = 1.32–15.76), p = 0.017]. Also, respondents who perceived high barriers to breast cancer screening were more likely to be screened for breast cancer [AOR = 0.15(95% CI = 1.42–4.22), p < 0.001].

**Conclusion:**

The uptake of screening among women of reproductive age in the Nandom Municipality is low. Health promotion interventions to improve breast cancer screening should target women with a tertiary level of education and should focus on heightening the perceived threat of breast cancer and minimizing barriers to breast cancer screening.

## Background

Breast cancer kills approximately 425,000 women and is the leading cause of cancer deaths amongst women globally [1]. There were over two million breast cancer cases in 2018 worldwide representing 15.3% of all cancer cases and 627,000 women died from the disease [1, 2]. The annual incident rate is 5.8% [3].

In Ghana, breast cancer remains a public health concern and the most common type of cancer among women in terms of incidence and mortality. Current epidemiological data on breast cancer are inadequate as most studies are based on clinic pathological characteristics [4–6]. However, interestingly, 30% of breast cancer cases in Ghana are below 35 years, which may indicate a relative possible shift of cancer burden to women in their early thirties compared to western countries [7].

Breast cancer screening is the cornerstone of early detection [8]. Four main screening modalities can be used to help detect breast cancer. The screening methods include breast self-examination (BSE), Clinical breast examination (CBE) (by a doctor or a nurse), Ultrasound of the breast and mammography. Breast cancer requires advanced facilities to diagnose and treat, which are mainly by surgery, chemotherapy, and radiotherapy which are done in few hospitals in the urban areas of Ghana. This creates a geographical barrier to accessibility for most Ghanaian women [9]. With the National Health Insurance Scheme not at its best, financial barriers to breast cancer screening thus exist, amongst other factors. Since breast cancer is a complex and heterogeneous disease with ethnic and social variations [10], each District or population in Ghana must have accurate knowledge that defines the characteristics of the disease amongst its people to determine ways of controlling its mortality. Many factors however are bound to influence breast cancer screening amongst women of reproductive age.

The Nandom Municipality has had its fair share of this galloping cancer as it is estimated according to end of year review that, out of every ten women who report to the various Maternal and Child Health Clinics in the various sub-Municipalities, at least one has a complaint of breast swelling, lumps or sore nipples, which are signs of breast cancer [11]. Unfortunately, because mammography is not done in the municipality women have to be referred to as far as to Tamale Teaching Hospital for the screening. Due to this breast cancer screening is a challenge in the municipality.

It is imperative therefore to unearth the factors that influence breast cancer screening in the Nandom Municipality to find ways of detecting and diagnosing the disease early to be able to stand a chance of curbing its menace. This can be achieved through systematic and thorough research. Nandom Municipality has no detailed literature on breast cancer screening so this study came out with some literature. Guided by the Health Belief Model (HBM), this study determined the factors influencing breast cancer screening among women in the Nandom Municipality of Ghana.

The current study was grounded on the HBM [12]. The HBM is a psychosocial model, which is widely used in health education and promotion. The idea of the HBM is that an individual’s health behavior is determined by his/her beliefs or perceptions about the disease and available plans to reduce the incidence of the disease [12].

The model focuses on six main constructs; perceived susceptibility, perceived seriousness, perceived barriers, perceived benefits, self-efficacy, and cues to action [12]. These constructs together determine a person’s likelihood of partaking in screening practices.

## Methods

### Study site

Nandom Municipality is one of the five Municipalities in the Upper West Region. The Municipality is located in the North-Western corner of the Upper West Region of Ghana between latitude 5° 18 W to 50° 10 W longitude of 1° 20 N to 2° 25 N. It shares boundaries with the Lambussie-Karni District to the East and Lawra District to the South and Burkina Faso to the North. The population of the Municipality in the 2010 population census stood at about 56,089 people with about 53.7% being females [13]. This showed the need to assess the factors that influenced the screening of breast cancer among women of reproductive age.

### Study population

The study included women of reproductive age (18–49) in the Nandom Municipality.

### Inclusion and exclusion criteria

All women of reproductive age and from Nandom Municipality who were available and consented to be part of the study. Those who fell within the inclusion criteria but who were seriously sick and admitted to a health facility were excluded.

### Study design

A quantitative study approach using a descriptive cross-sectional design was employed in this study using a survey-type questionnaire.

### Sample size determination and sampling method

The sample size was calculated using the formula by Degu and Tessema (2005) [14]. It was calculated based on 21.1% CBE among nurses in Ghana [15]. Assuming a Z score of 1.96 for a 95% level of confidence and a 5% margin of error, the sample size was 243.

$n=\frac{{Z(\alpha /2)}^{2}p(1-p)}{{e}^{2}},$ where n = sample size.

Zα/2 = Z score of 1.96 at 95% confidence interval (CI).

P = proportion of women of higher risk of breast cancer in Ghana, 21.2%

e = margin of error, 5%

n = $$\frac{{\left( {1.96^{2} } \right)*0.2*\left( {1 - 0.212} \right)}}{{0.05^{2} }}$$

n = 243.

The study respondents were selected using a multistage sampling technique. The Nandom Municipality has five (5) sub-Municipalities, which are Ketuo, Ko, Baseble, Gengenkpe, and Nandom. To get a true representation of the Municipality, participants were selected evenly from each of the five sub-Municipality to participate based on consent. In each sub-Municipality, the names of all the communities were written on pieces of paper. These were mixed up and one community was picked randomly. A convenience sampling technique was then used to select participants. The researcher visited the child welfare clinic (CWC) of the selected communities from each sub-Municipality to administer the questionnaire to 49 women in each of the communities. The purpose of the study was explained to the nurses at the CWC by the research team. The nurses explained the purpose of the study to the women who brought their children for CWC services and directed them to the study team. The purpose of the study was further explained to the mothers by the research team. Participant information and consent forms (PICFs) were given to the participants and the study team members were available to answer questions raised by the participants. Those who consented to participate were selected for the study.

### Data collection procedure

The data were collected by the use of a standardized questionnaire in October 2021. The completion of the questionnaires was done in the participants’ homes and the CWCs. Data were collected from participants who consented to participate in the study. Two persons at the various sub-Municipalities were trained to assist the researcher in the data collection. A pretested structured questionnaire adapted from Aba (2019) [16] was used to collect data on socio-demographic characteristics, uptake of breast cancer screening and factors that influence breast cancer screening based on the constructs of the HBM. At the end of every data collection session, the research assistants and the principal researcher reviewed all the questionnaires for accuracy and completeness before they were placed in files. This process continued till the sample size for the study was attained.

## Measures

The uptake of breast cancer screening (dependent variable) was measured by using the number of people who have ever screened for breast cancer and those who have never screened. Additionally, composite scores were generated for the constructs of the HBM (independent variables). For all the constructs of the HBM, the responses ‘Strongly agree’ and ‘Agree’ were combined into ‘Agree’. Also, the responses ‘Strongly disagree’ and ‘Disagree’ were combined into ‘Disagree’. On the construct perceived susceptibility, 3 items were used: ‘I am likely to get breast cancer’, ‘The chances of getting breast cancer in the next few months are high’ and ‘I feel I will get breast cancer at a point in life’. Furthermore, the number of items used to measure perceived severity and perceived benefits were 3 (breast cancer can lead to death, breast cancer can lead to cutting of the beast & breast cancer is very dangerous to all women) and 6 (my family will benefit if I screen for breast cancer, I am not worried if nothing is found, screening can detect lumps early, treatment could be easier if a lump is detected early, screening is the best way to find a very small lump & screening can reduce my chances of dying from breast cancer) respectively. Twelve items were used to measure perceived barriers (I do not understand the screening procedure, I am afraid of a positive result, I do not know how to go about getting screened for breast cancer, It is too embarrassing to be screened, screening procedure takes too much time, health workers doing the screening are rude to patients, screening will expose me to unnecessary radiation, it is difficult to schedule a breast cancer screening, breast cancer screening is not a priority to me, I am too old to need a routine breast cancer screening, breast cancer screening is too painful & breast cancer screening is expensive) while 9 items measured self-efficacy (I can arranged for transportation to get screened for breast cancer, I can create time to have a breast cancer screening, I am confident to talk to staff at the screening center about my concerns, I have the confidence to go for breast cancer screening, I can afford the cost for breast cancer screening, I can schedule an appointment for breast cancer screening, I can always go for screening if I want to, I know how to go about getting screening for breast cancer, & I can locate a breast cancer screening center). Composite scores were generated from the responses using the 50th percentile (median). This was done to generate a binary composite score for each construct. The median was therefore used as the point to categorise the respondents into those with low and high perceived susceptibility, severity, benefits, barriers and self-efficacy.

### Statistical analysis

The collected data were entered into EPI Data version 4.0.2.101 and exported into the STATA version 16 for analysis. Descriptive statistics such as frequencies and percentages were used to summarise the data. Binary logistic regression analysis was used to determine the association between breast cancer screening uptake and the independent variables (demographic variables and the constructs of the HBM) at the 0.05 level of significance and at a 95% confidence interval.

## Results

### Demographic characteristics of respondents

From Table 1, the majority 138(56.8%) of the respondents were aged less than 30 years; 69(28.4%) had a Junior High School (JHS) level of education and the majority 211(86.8%) were Christians. The majority 189(77.8%) were married and 138(56.8%) were housewives.

**Table 1:** Demographic characteristics of respondents (N = 243)

Variable	Frequency (Percentage) N(%)
*Age group*	
Less than 30	138(56.8)
30–39	77(31.7)
40 and above	28(11.5)
*Level of education*	
No formal education	49(20.2)
Primary	49(20.2)
JHS	69(28.4)
SHS/Technical	41(16.9)
Tertiary	35(14.3)
*Religion*	
Christianity	211(86.8)
Islamic	30(12.4)
Traditional	2(0.8)
*Marital status*	
Never Married	54(22.2)
Married	189(77.8)
*Occupation*	
Civil Servant	25(10.3)
Housewife	138(56.8)
Others	26(10.7)
Unemployed	54(22.2)

*JHS* junior high school; *SHS* senior high school

### Uptake of breast cancer screening

The majority of the respondents 126(51.9%) had ever screened for breast cancer. Also, only 35(14.4%) had ever had their breast examined by a healthcare provider (CBE). To add, 56(23.0%) of the respondents had been screened more than a year ago and 29(11.9%) had their screening between 3 and 6 months ago (Table 2).

**Table 2:** Uptake of breast cancer screening by respondents

Variable	Frequency (Percentage) N(%)
*Screened for breast cancer*	
Screened	126(51.9)
Never screened	117(48.1)
*The breast cancer screening method used*	
Clinical breast exam	35(14.4)
Breast-self exam	82(33.7)
Mammogram	6(2.5)
Ultrasound	3(1.2)
None	117(48.2)

### Association between demographic characteristics and breast cancer screening

The associations between the demographic characteristics of respondents and breast cancer screening uptake are presented in Table 3. Respondents who had a tertiary level of education were less likely to be screened for breast cancer [AOR = 0.10 (95% CI = 0.02–0.54); p = 0.008].

**Table 3:** Association between demographic characteristics and uptake of breast cancer screening

Variable	Uptake of breast cancer screening		Chi-square (p-value)	COR (95% CI) (p-value)	AOR (95% CI) (p-value)
	Screened n (%)	Never screened n (%)			
*Age group*					
Less than 30	78(56.5)	60(43.5)			
30–39	36(46.8)	41(53.2)			
40 and above	12(42.9)	16(57.1)	2.91(0.233)		
*Educational level*					
No formal education	17(34.7)	32(65.3)		Ref	Ref
Primary	28(57.1)	21(42.9)		0.40(0.03, 0.18) (0.027)	0.46(0.20, 1.08) (0.074)
JHS	26(37.7)	43(62.3)		0.88(0.41, 1.89) (0.740)	1.16(0.50, 2.70) (0.737)
SHS/Technical	25(61.0)	16(39.0)		0.34(0.14, 0.80) (0.014)	0.48(0.18, 1.30) (0.151)
Tertiary	30(85.7)	5(14.3)	29.32(< 0.001)	0.09(0.03, 0.27) (< 0.001)	**0.10(0.02, 0.54) (0.008)**
*Religion*					
Christianity	113(53.6)	98(46.4)			
Islamic	12(40.0)	18(60.0)			
Traditionalist	1(50.0)	1(50.0)	1.94(0.380)		
*Marital status*					
Never married	28(51.9)	26(48.1)			
Married	98(51.9)	91(48.1)	0.00(1.000)		
*Occupation*					
Civil servant	21(84.0)	4(16.0)		Ref	Ref
House Wife	62(44.9)	76(55.1)		6.44(2.10, 19.74) (0.001)	0.97(0.15, 6.45) (0.973)
Others	12(46.2)	14(53.8)		6.13(1.64, 22.89) (0.007)	0.86(0.11, 6.47) (0.883)
Unemployed	31(57.4)	23(42.6)	14.01(0.003)	3.89(1.18, 12.90) (0.026)	0.62(0.09, 4.47) (0.636)

### Constructs of the health belief model

Overall, the majority of the respondents 159(65.4%) perceived that they were susceptible to breast cancer; most 225(92.6%) believed that breast cancer is a serious condition; the majority (96.3%) believed that breast cancer screening is beneficial; most 138 (56.8%) perceived some barriers prevented them from getting screened for breast cancer and the majority 227 (93.4%) had a high self-efficacy for breast cancer screening (Table 4).

**Table 4:** Constructs of the health belief model

Constructs	Frequency (Percentage) N(%)
*Perceived susceptibility score*	
Low Susceptibility	84(34.6)
High Susceptibility	159(65.4)
*Perceived severity score*	
Low severity	18(7.4)
High severity	225(92.6)
*Perceived benefit score*	
Low benefit	9(3.7)
High benefit	234(96.3)
*Perceived barriers*	
Low barriers	105(34.2)
High barriers	138(56.8)
*Perceived self-efficacy*	
Low self-efficacy	16(6.6)
High self-efficacy	227(93.4)

### Logistic regression of factors influencing breast cancer screening based on the HBM

The associations between the uptake of breast cancer screening and the constructs of the HBM are shown in Table 5. Respondents who perceived high susceptibility to breast cancer were more likely to be screened for it [AOR = 1.97 (95% CI = 1.12–3.47), p = 0.019]. Respondents who perceived the high severity of breast cancer were more likely to be screened for it [AOR = 4.55 (95% CI = 1.32–15.76), p = 0.017]. Also, respondents who perceived high barriers to breast cancer screening were less likely to be screened for it [AOR = 0.15(95% CI = 1.42–4.22), p < 0.001].

**Table 5:** Logistic regression of factors influencing breast cancer screening based on the HBM

Variable	Uptake of breast cancer screening		Chi-square (p-value)	COR (95% CI) (p-value)	AOR (95% CI) (p-value)	Pseudo R2
	Screened n(%)	Never screened n(%)				General Pseudo R2 = **7.35%**
*Perceived susceptibility*						
Low susceptibility	54(64.3)	30(35.7)		Ref	Ref	
High susceptibility	72(45.3)	87(54.7)	7.95(0.005)	2.18(1.26, 3.75) (0.005)	**1.97(1.12, 3.47) (0.019)**	**2.39%**
*Perceived severity*						
Low severity	14(77.8)	4(22.2)		Ref	Ref	
High severity	112(49.8)	113(50.2)	5.23(0.022)	3.53(1.13, 11.06) (0.030)	**4.55(1.32, 15.76)(0.017)**	**1.65%**
*Perceived benefit*						
Low benefit	6(66.7)	3(33.3)				
High benefit	120(51.3)	114(48.7)	0.82(0.365)			
*Perceived barriers*						
Low barrier	67(63.8)	38(36.2)		Ref	Ref	
High barrier	59(42.8)	79(57.2)	10.59 (< 0.001)	2.36 (1.40, 3.98) (< 0.001)	**0.15(1.42, 4.22) (< 0.001)**	**3.18%**
*Perceived Self-efficacy*						
Low self-efficacy	10(62.5)	6(37.5)				
High self-efficacy	116(51.1)	111(48.9)	0.78(0.378)			

The overall predictive power of the HBM over breast cancer screening was 7.35%

## Discussion

This current study revealed that a majority (51.9%) of the respondents had ever screened for breast cancer. The study also reported that only 2.5% of the women had ever examined their breasts using a mammogram. Low rates of using mammography for breast cancer screening have been reported in similar studies conducted in Ghana [17–20]. Mammography is often used as a diagnostic examination rather than for screening because of the lack of routine screening mammography services and the high cost involved in Ghana [17]. The addition of mammography services to the Ghana National Health Insurance Scheme (NHIS) would be beneficial and would catalyze breast cancer screening.

Also, the current study revealed that only 14.4% of the respondents had undergone CBE. This result is very low as CBE is less expensive and effective in the detection of lumps and other abnormalities in the breast. Similarly, other studies conducted in Ghana reported low usage of CBE [17, 20, 21]. Further, an interventional study conducted in Kenya reported that the rate of CBE uptake among women increased by 38.0% as the intervention group received community-based health education from community health workers [22]. This indicates the need to create CBE awareness in the communities to improve upon the low uptake rate for early detection and treatment of cancer. The low uptake of CBE in Ghana is worrying as the survival rate of breast cancer is 39% [23]. The absence of a national cancer registry could also mean that the cases are under-reported. Furthermore, 33.7% of the respondents in the current study practised BSE. A slightly higher percentage (42.6%) of trainee health professionals in Ghana undergoing BSE has also been reported by Osei-Afriyie et al. (2021) [20]. Another study conducted among breast cancer patients in Ghana revealed that respondents rarely performed BSE before their diagnosis [24].

The current study revealed that respondents who had a tertiary level of education were less likely to be screened for breast cancer. However, a similar study conducted in Accra and Sunyani, Ghana, showed that a higher educational level was significantly associated with the uptake of breast cancer screening [17]. The finding from the current study could mean that women who have attained tertiary education might be overwhelmed with work schedules and not able to attend or book an appointment for breast cancer screening. Also, a study conducted in Ghana showed that respondents who had ever attended school were more likely to take up breast cancer screening [19]. Also, a study conducted among women in Iran showed that educational status was significantly associated with BSE [25].

The factors that may influence the uptake of breast cancer screening investigated in the current study include the constructs of the HBM (perceived susceptibility, perceived severity, perceived benefits, perceived barriers and self-efficacy). Concerning perceived susceptibility, the current study revealed that the majority (65.4%) of the respondents believed they were susceptible to breast cancer. In contrast, a study conducted in Ghana among female clinicians in Ga West and South revealed that 55.0% of the respondents had low perceived susceptibility to breast cancer [26].

Regarding the perceived severity of breast cancer, the current study revealed that the majority of the respondents (92.6%) believed that getting breast cancer would be dangerous. This is similar to a cross-sectional study conducted among students in Iran, which reported a high perceived severity of breast cancer [27]. From the current study, the belief that breast cancer could lead to death, cutting off the breast, and the danger of breast cancer accounted for the high perceived severity recorded. This implies that respondents would be more likely to take up breast cancer screening to prevent the seriousness of breast cancer. Health interventions should, therefore, heigthen the severity of breast cancer in health promotion interventions so that people can get screened for early detection and treatment.

Additionally, 96.3% of the respondents in the current study believed that breast cancer screening is beneficial. A comparable study conducted in Ghana reported similar findings [26]. In divergence, a study conducted Ghana in the Accra Metropolitan area among nurses and midwives revealed that 67.0% perceived breast cancer screening not to be beneficial [21].

About 56.8% of the respondents in the current study believed that there are barriers that prevent them from undertaking breast cancer screening. A similar study conducted in Ghana among female clinicians revealed that 51.0% of them perceived high barriers to breast cancer screening [26]. Some of the barriers stated by respondents in this current study include: fear to find out something is wrong (49.8%), and did not know where to get screened (40.9%). Also, 43.2% and 35.0% believed that the screening was painful and expensive respectively. These barriers need to be minimised or removed to encourage women to take up breast cancer screening. For instance, breast cancer screening could be covered by the NHIS so that women would not have to pay additional money to get screened if they visit the health facility.

On perceived self-efficacy, 93.4% of the respondents in the current study were confident that they could take up breast cancer screening. This implies that they believed they could overcome the existing barriers that exist and get screened for breast cancer. Similar results were found from a cross-sectional study conducted among students in Iran, which showed that perceived self-efficacy was high among the respondents [27]. This indicates that increasing the confidence of women toward breast cancer screening is, therefore recommended to improve the uptake of the services. Also, a study among clinicians in Ga West and South Districts of Ghana showed that about 54% of the respondents had low self-efficacy regarding breast cancer screening [26]. This further iterates the need to increase the confidence of women so that they can easily take up breast cancer screening.

The association between the constructs of the HBM and breast cancer screening uptake showed that respondents who perceived high susceptibility to breast cancer were more likely to be screened for breast cancer. Similarly, a study conducted in Turkey among women who were 40 years and above showed that perceived susceptibility was a strong predictor of breast cancer screening [28]. Also, a study among undergraduate students in the Volta Region in Ghana showed that those who did not believe to be susceptible to breast cancer were less likely to get screened [20]. It can be inferred from the current finding that designing interventions to target the perceived susceptibility of respondents is essential as it would make clear the risk factors of breast cancer.

Also, the current study revealed that respondents who perceived a high severity of breast cancer were more likely to be screened for the disease. The perceived seriousness of breast cancer was also found to be a strong predictor of breast cancer screening among older women in Turkey [28]. A similar study conducted among students in Northwest Iran showed that high perceived severity was a predictor of breast cancer screening behaviour [27]. The severity of breast cancer to the individual, the family and the society at large should be made a central point in health promotion interventions so that women would be compelled to get screened.

Furthermore, respondents who perceived high barriers to breast cancer screening were less likely to be screened for breast cancer. In consonance, a similar study conducted in Iran showed that perceived barriers were significantly associated with breast cancer screening [25]. A study conducted in Turkey reported that perceived barriers had a strong association with breast cancer screening [28]. Also, a similar study conducted among clinicians in Ghana showed that perceived barriers were significantly associated with the uptake of breast cancer screening [26]. In this current study, it can be said that respondents who perceive barriers could find it difficult to get screened for breast cancer. Health promotion interventions to improve breast cancer screening should focus on reducing barriers to screening.

The current results should, however, be interpreted in line with some limitations. The convenience sampling used at the last stage of the multistage sampling is a non-probability sampling method and may limit the generalizability of the findings of this research. The cross-sectional nature of the study design limits the ability to attribute a causal relationship between the factors associated with breast cancer screening and screening uptake among the participants. Also, the study used a questionnaire to elicit responses on a sensitive topic (breast cancer screening) that has the potential of introducing social desirability bias and there was no way to validate what the respondents reported. However, the assurance of anonymity and confidentiality of the responses should have minimized possible limitations. Cultural factors could also shape breast screening behaviour in the Ghanaian context, but these factors are not accounted for in the HBM. Despite these limitations, this study provides insight into the factors influencing breast cancer screening among women of reproductive age in the Nandom Municipality, Ghana using the HBM.

## Conclusion

The uptake of breast cancer screening among women of reproductive age in the Nandom Municipality was considerably low (51.9%). Health promotion interventions to improve breast cancer screening should target women with a tertiary level of education and should focus on heightening the perceived threat of breast cancer and minimizing barriers to breast cancer screening.

## Data Availability

The datasets used and/or analysed during the current study are available from the corresponding author upon reasonable request.
